# End-of-life care in Germany: Study design, methods and first results of the EPACS study (Establishment of Hospice and Palliative Care Services in Germany)

**DOI:** 10.1186/1472-684X-9-16

**Published:** 2010-07-30

**Authors:** Luis Carlos Escobar Pinzón, Eva Münster, Sabine Fischbeck, Michael Unrath, Matthias Claus, Tanja Martini, Martin Weber

**Affiliations:** 1 University Medical Center of the Johannes Gutenberg University of Mainz, Institute of Occupational, Social and Environmental Medicine, Obere Zahlbacher Straße 67, 55131 Mainz, Germany; 2 University Medical Center of the Johannes Gutenberg University of Mainz, Clinic of Psychosomatic Medicine and Psychotherapy, Medical Psychology and Medical Sociology, Saarstraße 21, 55099 Mainz, Germany; 3 University Medical Center of the Johannes Gutenberg University of Mainz, Interdisciplinary Palliative Care Unit, Langenbeckstraße 1, 55131 Mainz, Germany

## Abstract

**Background:**

In order to tackle the deficits in the provision of palliative home care, profound structural changes in the outpatient sector were introduced by law in Germany in 2007. The EPACS study was carried out (Research Accompanying the **E**stablishment of Hospice and **Pa**lliative **C**are **S**ervices in Rhineland-Palatinate, Germany) to document the quality of inpatient and outpatient end-of-life care in Rhineland-Palatinate, Germany, before the implementation of these changes. With this article we focus on the study design and methods of the EPACS-Study. We further report first results regarding several aspects of outpatient end-of-life care.

**Methods:**

The cross-sectional survey was based on a random sample of 5000 inhabitants of Rhineland-Palatinate that had died from May 25 until August 24 of the year 2008. Relatives of these randomly drawn deceased persons were interviewed by means of a written survey.

**Results:**

The overall response proportion considering only those questionnaires that actually were delivered (n = 3833) was 36.0%. Factors influencing participation were age, sex, and marital status. 355 (25.8%) deceased persons had used professional home care in the four weeks prior to their death, but only very few persons had used a specialised palliative home care service (n = 30; 8.5%). There was a clear gap between the need for specialised outpatient care and the actual utilisation of these services.

**Conclusions:**

Satisfaction with professional home care was relatively high, but physicians were rated less favourable than nurses. There were deficits especially with respect to physicians' communicative and supportive skills. Further analyses are necessary to provide more detailed information about quality of care in different care settings and for distinct groups. Predictors of good care, as well as obstacles to it, must be further investigated. In the long run, a follow-up survey must be conducted to compare quality of home care before and after the structural changes in Germany.

## Background

Palliative care has been defined by the World Health Organization as an approach to improve the quality of life of patients who face life-threatening, chronic diseases. Pain relief and treatment of other physical or psychosocial problems are among the main goals of palliative care. Palliative care should not only address the patients' needs, but also the requirements of their families and caregivers. Physical as well as psychological, social and spiritual aspects of care are core elements of this approach [1]. With the rise of chronic diseases and prolongation of life expectancy in Western European countries, palliative care performed by professional carers already is important today and will presumably gain importance in the years to come. There will be a growing number of patients suffering from serious chronic conditions. At the same time, changes in the age structure will cut back the potential of families and other informal caregivers for adequate nursing care. The existing health care systems will have to be adapted to cope with these new challenges [2].

Palliative care structures in Western European countries evolved at different paces and levels of intensity [3]. In Germany, palliative care structures have been developed both in the inpatient and the outpatient sector, but these current structures are still insufficient [4]. This especially applies to the outpatient sector. Until recently, only a tiny fraction of the necessary professional palliative homecare services existed. A nationwide survey in 2004 showed a number of 1100 ambulatory hospice services based mainly on the commitment of volunteers compared with only 35 specialised palliative homecare services [5]. Further on the availability of palliative care services in Germany is characterised by considerable regional differences with very well developed services in some urban areas in contrast to extensive gaps in rural areas [6,7].

Since numerous studies have shown that the vast majority of patients wish to die at home, this obvious deficit in the home care sector is even more worrying [8-11]. Moreover quality of life and satisfaction of patients are generally rated more favourable when people are cared for in their homes with help of specialised palliative care teams [12-16]. Finally, homecare seems also to be superior to inpatient care with respect to public expenditures in the health care system [17,18].

In order to tackle the deficits in the provision of palliative home care, profound structural changes in the outpatient sector were introduced by law in Germany in 2007 [19]. With this new law, specialised outpatient palliative care services are rendered possible nationwide. With the new law every patient in need has the right to receive specialised palliative home care. Out of this an obligation to reimburse this service arises for the health insurances. Currently this reimbursement is negotiated in many regions in Germany [20]. The practical implementation of this law was expected to start by the end of the year 2008 after the framing of the corresponding guide-lines.

In order to evaluate the effects of these profound structural changes, accompanying research is necessary. Will the structural changes really improve the situation of patients and families? How will quality of care and satisfaction with care develop? To answer these questions, the situation as it was before the structural changes must be documented in a first step. The overall aim of the EPACS study (Research Accompanying the **E**stablishment of Hospice and **Pa**lliative **C**are **S**ervices in Rhineland-Palatinate, Germany) is therefore to gain a representative picture of end-of-life inpatient and outpatient care in the federal state of Rhineland-Palatinate, Germany, as it was before the introduction of new specialised palliative home care structures.

We intended to identify unfulfilled needs of patients and relatives, utilisation of specialist palliative care services, existing gaps and satisfaction with different settings of end-of-life care.

With this article we focus on the study design and methods of the EPACS-Study, and analyse factors that could have influenced participation in our study. We further report first results regarding several aspects of outpatient end-of-life care, as these are of special interest in the light of the upcoming structural changes.

## Methods

**Figure 1:**
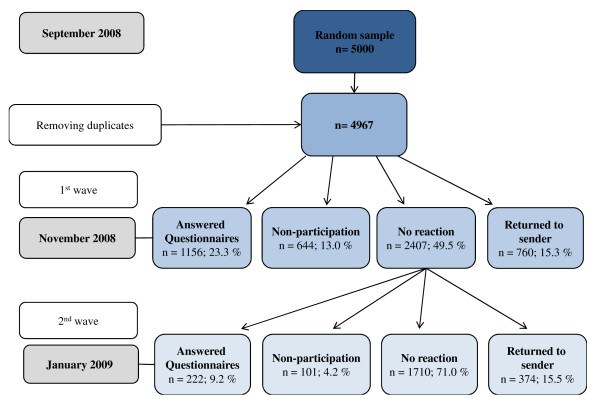
**Data collection of the EPACS study**.

Our cross-sectional study was carried out between September 2008 and January 2009 by the Institute of Occupational, Social and Environmental Medicine, the Interdisciplinary Palliative Care Unit, and the Medical Psychology and Medical Sociology, Clinic of Psychosomatic Medicine and Psychotherapy, University Medical Center of the Johannes Gutenberg University of Mainz. The written survey addressed relatives of deceased people with principal residence in Rhineland-Palatinate, Germany. Figure 1 illustrates the whole process of data collection.

### Provision of the sample

The ethical committee of the medical association of the German State of Rhineland-Palatinate and the data protection officer of Rhineland-Palatinate approved the EPACS study. It is conform to the actual Declaration of Helsinki on Ethical Principles for Medical Research. After permission of the ministry of the interior and the data protection commissioner, a random sample of 5000 addresses of deceased inhabitants was drawn from all local registry offices in the federal state of Rhineland-Palatinate. The requested random sample was drawn from all inhabitants that had died from May 25 until August 24 of the year 2008 and had their first residence in Rhineland-Palatinate. According to the Regional Bureau of Statistics 10183 persons died in Rhineland-Palatinate during this period of time. Therefore our random sample consisted of 49.1% of all deceased. The exact dates of death were not revealed for data protection reasons. Instead, time intervals were defined. Interval 1 reached from May 25 until June 24, interval 2 from June 25 until July 24, and interval 3 from July 25 until August 24. Duplicates had been deleted. Apart from the names, addresses and time intervals, further information about the deceased persons was provided: Sex, nationality, age (in years), marital status, and official community code of the principal residence.

### Data collection

The random addresses were used to contact the deceased inhabitants' next relatives via postal mail at the end of September 2008. In our cover letter, they were asked to fill in a standardised and pseudonymised questionnaire. This questionnaire was accompanied by detailed instructions on a separate page and a declaration of non-participation which should be filled in and sent back in case the relative did not want to participate. Non-participants were asked to indicate the reason of non-participation by means of several anticipated answers (e.g. "emotionally too draining") or by free-text [additional file 1]. The rather short interval from death to time of posting the questionnaire (between 1 month and 4 months) was chosen to ensure forwarding of the sending in case that no family members lived at the address of the deceased. Furthermore we aimed to reduce recall bias. In November 2008 we sent reminder letters to those addresses, where no reaction had been registered so far. Due to ethical considerations, no further efforts were made to contact the relatives after this first reminder letter. Data collection and statistical processing of the data were carried out at the Institute of Occupational, Social and Environmental Medicine, University Medical Center of the Johannes Gutenberg University of Mainz.

### Questionnaire

For our survey we developed a specific questionnaire. The questionnaire focused on questions referring to the type and quality of inpatient and outpatient care during the last four weeks before death. Issues concerning the care for the bereaved relatives after death were addressed as well. The questionnaire, amongst others, contained questions from a module for relatives after the patient's death which is offered within the Hospice and Palliative care Evaluation (HOPE), a standardised basic documentation tool developed and evaluated by a multiprofessional working group since 1996, which has been used in several epidemiologic surveys in Germany [21,22]. The questionnaire also included questions about general satisfaction with care [23].

Further batteries of questions related to the underlying diseases and the extent of care needed by the deceased person, socio-demographic and socio-economic parameters, and the personal situation of the relative. The questions regarding the type of care and the underlying diseases mostly allowed more than one answer (multiple choice questions) [additional file 2]. Altogether, the questionnaire covered 63 questions, most of which consisted of several standardised items.

The questionnaire was tested in terms of feasibility and was optimised in a pilot survey (n = 12) directed at selected relatives of deceased patients that had been treated in different settings (home care, the palliative care unit of the University Medical Center of the Johannes Gutenberg University of Mainz, the inpatient Hospice of Mainz and an affiliated nursing home). It took participants about 30-40 minutes to fill in the questionnaire. There were no difficulties in understanding the questions.

### Statistical analysis

All analyses were carried out with SPSS version 17.0. An error probability of α = 0.05 was assumed for all statistical tests.

#### Descriptive statistics

The representativeness of the random sample was tested statistically by means of Chi-Square Goodness-of-Fit Tests.

In the first analysis of the data presented here, absolute and relative frequencies were calculated for the general description of the socio-demographic variables and the variables relating to quality and extent of professional home care. Moreover, mean, median and standard deviation were calculated for the variable age. The variable age was also categorised into age groups that had been defined a priori. The first age group comprised all individuals younger than 40 years. The following age categories comprised 10 years each (40-49, 50-59 and so on). The last age group included individuals that were 90 years or older.

#### Bivariate analysis

Bivariate associations between socio-demographic variables such as age, sex, and nationality and the variable "reaction" (to the questionnaire) were analysed by means of Chi Square Tests.

#### Multivariate analysis

A considerable share of the questionnaires could not be delivered to the addresses we had obtained from the local registry offices. This was probably due to address changes which occurred relatively fast after the death of the resident.

As we particularly wanted to contrast the deliberate decision to participate with the decision not to participate, we created a dichotomous variable "type of participation" which contained those relatives who participated in the survey and those who decided not to participate even though they had received a questionnaire. This was achieved by recoding the variable "reaction", which originally comprised the four categories "participation", "non-participation", "no reaction" and "returned to sender". We kept the category "participation", but excluded the category "returned to sender". The other two categories ("non-participation", "no reaction") were combined into a new category "no participation".

In order to estimate the independent effects of the socio-demographic variables sex, age, marital status and nationality on the "type of participation" (decision to participate vs. decision not to participate), a binary logistic regression model (inclusion method) predicting participation was calculated. Adjusted Odds Ratios and the corresponding 95%-Confidence Intervals were estimated.

## Results

### Random sample

Comparisons with the death statistics of the federal state of Rhineland-Palatinate showed that the random sample was representative regarding the distribution of age, sex and nationality among all deaths occurring in the respective period of time (n = 10183).

The random sample consisted of 2324 (46.8%) deceased men and 2643 (53.2%) deceased women. Their age ranged from babyhood to over 100 years and was distributed around a mean age of 77.0 years (median = 80.0 years, SD = 14.1). The age distribution was clearly left skewed with 76.3% of the deceased being 70 years or older. 2066 (41.6%) and 2121 (42.7%) deceased had been either married or widowed. Relatively few deceased had been divorced (n = 310; 6.2%) or never been married (n = 469; 9.4%). The deceased's nationality was mainly German (n = 4886; 98.4%). Only 1.6% (n = 81) had a foreign nationality.

### Overall participation

Altogether, 1378 (27.7%) of the 4967 valid questionnaires were answered. For further 745 questionnaires, a declaration of non-participation was sent back. The overall rate of return considering only those questionnaires that actually were delivered (n = 3833) was 36.0%.

Details of data collection are shown in figure 1.

### Reactions to the questionnaire

#### Bivariate analysis

Absolute and relative frequencies of the types of reaction stratified by the categories of the socio-demographic variables are displayed in table 1.

**Table 1:** Types of reaction to the questionnaire of the EPACS study stratified by the socio-demographic characteristics of the deceased, n = 4967

	Total	Answered questionnaires		Non-participation		No reaction		Returned to sender		p value
	n	n	%	n	%	n	%	n	%	
**Sex**										**< 0.001**
Male	2,324	612	26.3	440	18.9	893	38.4	379	16.3	
Female	2,643	766	29.0	305	11.5	817	30.9	755	28.6	
**Marital Status**										**< 0.001**
Married	2,066	654	31.7	436	21.1	844	40.9	132	6.4	
Widowed	2,121	594	28.0	224	10.6	630	29.7	673	31.7	
Divorced	310	47	15.2	25	8.1	85	27.4	153	49.4	
Unmarried	469	83	17.7	60	12.8	150	32.0	176	37.5	
**Nationality**										**0.16**
German	4,886	1,364	27.9	733	15.0	1,675	34.3	1,114	22.8	
Other	81	14	17.3	12	14.8	35	43.2	20	24.7	
**Age**										**< 0.001**
0 - 39 years	85	14	16.5	21	24.7	35	41,2	15	17.6	
40 - 49 years	153	37	24.2	13	8.5	61	39.9	42	27.5	
50 - 59 years	307	84	27.4	37	12.1	132	43.0	54	17.6	
60 - 69 years	631	185	29.3	114	18.1	223	35.3	109	17.3	
70 - 79 years	1,187	322	27.1	219	18.4	469	39.5	177	14.9	
80 - 89 years	1,904	526	27.6	259	13.6	627	32.9	492	25.8	
≥ 90 years	700	210	30.0	82	11.7	163	23.3	245	35.0	

The reactions to the questionnaire differed significantly according to the sex of the deceased person. More relatives of deceased women answered the questionnaire than relatives of deceased men (n = 766; 29.0% vs. n = 612; 26.3%; p < 0.001). On the other hand, nearly twice as many questionnaires of deceased women were returned to sender in contrast to questionnaires of deceased men (n = 755; 28.6% vs. n = 379; 16.3%; p < 0.001).

The deceased's marital status showed similar statistically significant differences with regard to the type of reaction to the questionnaire (p < 0.001). Twice as many questionnaires concerning married (n = 654; 31.7%) or widowed persons (n = 594; 28.0%) were received in comparison with questionnaires relating to divorced (n = 47; 15.2%) and unmarried persons (n = 83; 17.7%).

The age of the deceased was also significantly associated with the type of reaction to the questionnaire (p < 0.001).

Relatives of German citizens did not differ significantly from relatives of foreigners with respect to their reaction to the questionnaire (p = 0.16). However, there was a tendency that fewer relatives of foreigners answered the questionnaire than relatives of German deceased. There were no significant differences (p = 0.18) between the time intervals in which the persons had died either.

#### Multivariate analysis

In our multivariate model predicting participation, similar associations as in the bivariate analysis could be observed. As can be seen from table 2, female sex of the deceased person was a significant predictor of participation. In contrast, relatives of unmarried persons were significantly less likely to participate than relatives of married persons. As to age, relatives of persons that died at an earlier age were less likely to participate than relatives of persons that died when they were already 90 years or older. The biggest of these effects occurred when contrasting the highest age group (≥ 90 years) with the lowest age group (< 40 years). Nationality failed marginally to become significant (p = 0.06), but there was a tendency in the sense that relatives of foreigners participated less often than relatives of German citizens.

**Table 2:** Binary logistic regression model predicting participation (vs. no participation) to the questionnaire of the EPACS study, n = 3832

Predictor	Category	n	**aOR**^**a**^	**95%-CI**^**b**^	P value
**Marital status**	Married (Ref.^c^)	1,934	-	-	-
	Widowed	1,448	1.1	0.9 - 1.3	.500
	Divorced	157	0.8	0.5 - 1.1	.174
	Unmarried	293	0.7	0.6 - 1.0	**.044**
**Age (in years)**	≥ 90 (Ref.)	455	-	-	-
	80 - 89	1,412	0.7	0.6 - 0.9	**.006**
	70 - 79	1,009	0.6	0.5 - 0.8	**< .001**
	60 - 69	522	0.8	0.6 - 1.0	**.049**
	50 - 59	253	0.7	0.5 - 1.0	**.048**
	40 - 49	111	0.8	0.5 - 1.2	.226
	< 40	70	0.4	0.2 - 0.8	**.008**
**Sex**	Male (Ref.)	1,944	-	-	-
	Female	1,888	1.4	1.2 - 1.6	**< .001**
**Nationality**	German (Ref.)	3,771	-	-	-
	Other	61	0.6	0.3 - 1.0	.063

^a^aOR = adjusted Odds Ratio

^b^CI = Confidence Interval

^c^Reference category

#### Declarations of non-participation

The most common reason not to participate reported in the 745 declarations of non-participation was that the topic was regarded as too emotionally draining (n = 389; 52.2%). 30.6% (n = 228) stated not to participate in surveys in general, and 4.0% (n = 30) reported that they had no time to answer the questionnaire. Other reasons not to participate were for example a sudden death (n = 29; 3.9%) or suicide (n = 7; 0.9%) of the relative. Furthermore, some non-participants had the feeling that the questionnaire was not matching their specific circumstances (n = 12; 1.6%), or had no contact with the deceased person (n = 17; 2.3%). 70 persons (9.4%) indicated no reasons why they did not want to participate.

### Sample of answered questionnaires

#### Socio-demographic variables

The distribution of socio-demographic variables in the sample of answered questionnaires (n = 1378) is displayed in table 1. A similar age distribution could be observed as in the random sample. 76.8% (n = 1058) of the deceased were 70 years or older, and just 1.0% (n = 14) were 40 years or younger. The mean age of the deceased was 77.6 years (median = 80, SD = 13.2). The sample consisted of more deceased females (n = 766; 55.6%) than males (n = 612; 44.4%). There were hardly any deceased without German nationality in the sample (n = 14; 1.0%). With regard to marital status, deceased persons who had been married (n = 654; 47.5%) and widowed (n = 594; 43.1%) clearly formed the two biggest groups, while relatively few deceased had either been unmarried (n = 83; 6.0%) or divorced (n = 47; 3.4%).

#### Evaluation of professional home care

The distribution of health-related variables for the 355 (25.8%) persons who made use of a home care nursing service in the four weeks prior to their death and for the whole sample of answered questionnaires is depicted in table 3.

**Table 3:** Distribution of health-related variables for persons who used a professional home care service (n = 355) and the whole sample of answered questionnaires (n = 1378) of the EPACS study

	Used professional home care		Answered questionnaires	
	n	%	n	%
**Proceeding, incurable, lethal disease**				
Yes	319	89.9	998	72.4
No	30	8.5	351	25.5
Missing	6	1.7	29	2.1
**Type of disease**a				
Cancer	112 (175)	31.6 (49.3)	334 (537)	24.2 (39.0)
Dementia	35 (99)	9.9 (27.9)	122 (310)	8.9 (22.5)
Cardiovascular diseases	18 (114)	5.1 (32.1)	115 (424)	8.4 (30.8)
Other	32 (146)	8.9 (41.1)	166 (554)	12.0 (40.2)
Multimorbidity (e.g. Cancer + Dementia + CVD)	151	42.5	504	36.6
Missing/I don't know	7	2.0	137	9.9
**Place of death**				
At home	229	64.5	526	38.2
In a hospital	92	25.9	541	39.3
In a palliative care facility	23	6.5	103	7.5
In a nursing home	11	3.1	185	13.4
Elsewhere	0	0.0	22	1.6
Missing	0	0.0	1	0.1
**Duration of home care**				
No home care	0	0.0	574	41.7
< 1 week	59	16.6	59	4.3
1 - < 2 weeks	42	11.8	42	3.1
2 - < 3 weeks	30	8.5	30	2.2
3 - 4 weeks	224	63.1	224	16.3
Missing/I don't know	0	0.0	449	32.6

^a^Multiple answers were permitted regarding the underlying disease. The figures outside the brackets show the number/percentage of people having suffered only from the aforementioned particular disease. Persons having suffered from more than one disease prior to death were assigned to the category "multimorbidity". In contrast, the figures in brackets show the number/percentage of people having suffered from the aforementioned disease and potentially from one or more additional diseases. In this regard, the percentage values in brackets always refer to the total number of people (i.e. 355 or 1378 respectively) and are not to be interpreted as column percentages.

As indicated by their relatives, 89.9% (n = 319) of persons using home care suffered from a proceeding, incurable, lethal illness. 49.3% (n = 175), for instance, had cancer. The vast majority of people receiving professional home care, died at home (64.5%) or in hospital (25.9%). The figures for the whole population are 38.2% and 39.3% respectively. Most people used a home care nursing service for three weeks and more during the four weeks prior to death. 30 persons (8.5%) were supported by a specialised palliative care nurse in addition to the home care nursing service [24].

**Figure 2:**
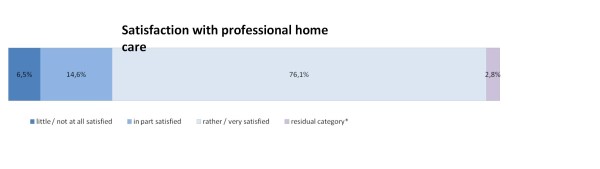
**Satisfaction with professional home care, n = 355**. *includes missing values and the category "not able to assess"

A majority of 76.1% (n = 270) of the participants were either rather or very satisfied in a general way with the professional home care their deceased relative had received. Only 6.5% (n = 23) were either little or not at all satisfied. Figure 2 displays the overall satisfaction with professional home care in the sample.

**Figure 3:**
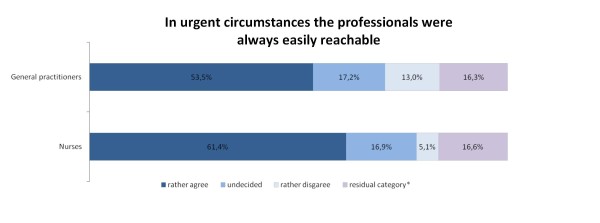
**Availability of professional home care in urgent circumstances, n = 355**. *includes missing values and the categories "professional was not involved" and "don't know"

With regard to more specific indicators of quality of care, both general practitioners and nurses were judged as being easily reachable in crises and as having enough time when needed by the majority of participants. Nurses were assessed slightly more positive (p = 0.003) than general practitioners with regard to availability in urgent circumstances, with 61.4% (n = 218) of the participants stating that they were easily reachable (vs. n = 190, 53.5% for the physicians). In contrast, a substantial percentage of participants (13.0%; n = 46) felt, that physicians were difficult to reach in urgent circumstances (5.1%; n = 18 for the nurses; p < 0.001). Figure 3 illustrates these findings.

Nurses also were assessed slightly more positive than general practitioners with regard to having enough time when needed (n = 218; 61.4% vs. n = 202; 56.9%; p = 0.275), whereas again an important percentage of participants judged that the physicians did not have enough time (11.0%; n = 39 vs. 7.9%; n = 28 for the nurses; p = 0.142).

**Figure 4:**
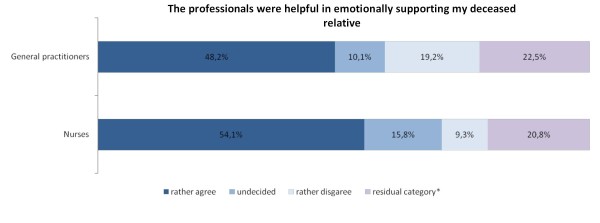
**Helpful emotional support in professional home care, n = 355**. *includes missing values and the categories "professional was not involved" and "don't know"

Concerning emotional support, participants again rated the nurses' performance somewhat more favourable in contrast to the physicians' performance (p = 0.032). Whereas 54.1% (n = 192) of the participants stated that the nurses were helpful in lending emotional support, only 48.2% (n = 171) reported the same for the general practitioners. 19.2% of the participants (n = 68) even stated that the physicians were not helpful at all regarding emotional support (vs. n = 33; 9.3% for the nurses; p < 0.001). Figure 4 gives a graphical overview of this result.

With regard to pain and symptom management, the majority of participants stated that pain (67.3%; n = 239) and other physical symptoms (54.4%; n = 193) such as nausea or shortness of breath were treated sufficiently. In line with this finding, 3.4% (n = 12), and 5.1% (n = 18), respectively, reported that pain and other symptoms were not treated sufficiently by the home care professionals.

**Figure 5:**
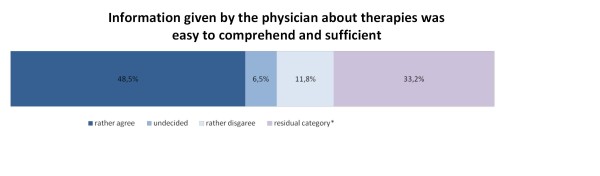
**Information given by the physician about therapies, n = 355**. *includes missing values and the categories "professional was not involved" and "don't know"

**Figure 6:**
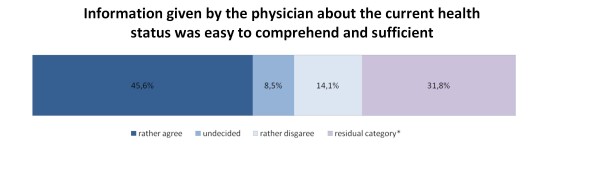
**Information given by the physician about the current health status, n = 355**. *includes missing values and the categories "professional was not involved" and "don't know"

As can be seen from figures 5 and 6, less than half of the participants rather agreed that the information given by the physician about therapies (48.5%; n = 172) and the current health status (45.6%; n = 162) was easy to comprehend and sufficient. 42 (11.8%) and 50 (14.1%) participants, respectively, rather disagreed that the quality of information given by the physician was good with respect to completeness and comprehensibility.

## Discussion

### Main findings

With our survey, we intended to gain a representative picture of end-of-life care in Rhineland-Palatinate, Germany, before the onset of profound structural changes in the outpatient sector introduced by law in Germany in 2007.

As has been shown, the random sample drawn by the local registry offices was representative of all deaths occurring in Rhineland-Palatinate in the year 2008. We reached a rate of return of 36.0%, which can be considered a satisfactory result taking into account the highly emotional topic of the survey and the detailedness of the questionnaire. There was only a moderate underrepresentation of foreign, unmarried, male or younger persons in our sample. The finding that participation - independent of marital status and age - was more likely if the deceased relatives were female is an interesting one and cannot be explained easily. Possibly male survivors tend to preserve a more rational coping behaviour and therefore may be somewhat more disposed to fill in a large questionnaire. The underrepresentation of citizens with foreign nationality can partly be explained by the fact that we only used German material for the survey. There are also possible explanations for the underrepresentation of younger and unmarried persons. The death of a young person might be more emotionally draining, because it occurs rather unexpected and unpredictable in contrast to the death of an older person. This assumption is supported by the fact, that there was the highest proportion of declarations of non-participation in the youngest age group. Unmarried persons, on the other hand, should on average have fewer close relatives that have the potential to actually participate. As can be seen from table 1, divorced and unmarried persons also had the highest proportion of questionnaires returned to sender, which leads to the assumption that many of these people were living alone or perhaps in a nursing home. There were no significant differences in the reactions to the questionnaire regarding the time interval in which the person had died. This is a bit surprising, because we expected a lower response rate for persons who had died shortly before the survey in contrast to persons whose death had been earlier in time. The opposite relationship was expected for questionnaires returned to sender. In contrast to these expectations, it seemed to be of minor importance for the reaction to the questionnaire if the death of the relative dated back four weeks or three months.

Concerning the evaluation of home care in the last four weeks prior to death, the majority of participants seem to agree that the professional caregivers were available when needed and had enough time. With respect to emotional support, the figures are less favourable, but overall, the picture seems to be quite positive. This positive picture is also supported by the finding that three out of four participants were rather or very satisfied with the quality of care. In interpreting these results, it has to be kept in mind that in satisfaction questionnaires, there is a tendency towards high satisfaction levels and ceiling effects [25,26]. The rather high proportion of home-deaths (64.5%) in this subgroup of patients receiving home care during the last 4 weeks can be interpreted as a further indicator of a helpful and supportive professional care in the majority of patients.

Looking to the results in more detail, it is striking that general practitioners were always rated less positive than nurses. This was especially true for emotional support. As to the communication between the physician and the patient, less than half of the patients agreed that the information given was sufficient and comprehensible. Since accurate and regular information as well as emotional support seem to play a significant role with respect to quality of life and satisfaction with care [27,28], these findings indicate a need for improvement. Indeed, our findings suggest a substantial percentage of home-care patients reaching up to 25%, whose physical and emotional needs are not sufficiently met by the established public health services. A possible approach could consist of specific training programmes designed to enhance supportive and communicative skills. The effectiveness of such programmes designed to improve the physicians' abilities regarding palliative home care has already been demonstrated [29]. Another important result is that only little use was made of specialised palliative home care services. Whereas almost 90% of those receiving professional home care suffered from an incurable, proceeding and life-shortening disease, only 8.5% of them were supported by specialist palliative care nurses. Here is an obvious gap between patients' needs and provision of specialised services. This gap should be filled by the ongoing structural changes, but needs further monitoring.

### Strengths and limitations

The need for more systematic scientific evaluation in the field of palliative care has been put forward by several authors, especially in conjunction with interventions on the public health level [30,31]. In this sense, the EPACS study is probably one of the first large-scale studies in Germany that seeks to determine the impact of structural health care interventions on the quality of end-of-life care. One of the special strong points is that we did not use a convenience sample of people treated in an inpatient facility or specialised programme, but a sample designed to be representative of a whole federal state [32]. Another strong point is that we collected a very broad and unique range of information including satisfaction with care as well as data on underlying diseases, type and extent of care, place of death, quality of dying, the personal situation of the relative, unfulfilled needs and wishes, and socio-demographic and socio-economic parameters.

The quality of our data is partly limited by the fact, that the questionnaire used (including the questions taken from the HOPE-module) was not tested for its psychometric properties. However, since our study was an exploratory approach primarily aimed at gathering basic information, the questionnaire was not conceived as a tool with defined psychometric properties, but rather as a first step in the assessment of different aspects in outpatient and inpatient end-of-life care.

Another limitation relates to the method of interviewing relatives of deceased persons. The possibility remains that judgements made by the relatives in our survey might deviate to some extent from what the deceased persons themselves would have answered. The direction and size of this deviation cannot be determined from the inconsistent literature [33,34]. Nevertheless, the method of interviewing relatives or caregivers of deceased persons has often been applied in epidemiological studies gathering information on representative samples [35]. In general, there seems to be a consensus that this method is valid in capturing quality aspects of life, death and care experienced by the deceased persons [34,36].

Finally another possible source of bias that always exists in retrospective studies is recall bias. In order to reduce possible systematic errors due to memory, we tried to conduct our survey as prompt as possible after the time of death without unnecessarily pressuring or molesting the bereaved relatives. Further analysis of the data will reveal if the evaluation of quality of care and satisfaction differs systematically across time intervals.

## Conclusions

We attained a sample of deceased persons suitable to draw generalisable conclusions for Rhineland-Palatinate, Germany. A broad range of useful information was collected to evaluate the outpatient palliative care in Germany.

Only few persons used a specialised palliative home care service in our sample. There was a clear gap between the need for specialised outpatient care and the actual utilisation or existence of these services. All in all, the satisfaction with professional home care was relatively high, but doctors were rated less favourable than nurses. There were deficits especially with respect to physicians' communicative and supportive skills, which could be tackled by appropriate programmes.

Further analyses are necessary to provide more detailed information about quality of inpatient and outpatient care in different care settings and for distinct groups. Predictors of and obstacles to good care must be further investigated. In the long run, a new survey must be undertaken to compare specific indicators of quality of care before and after the structural changes in Germany.

## Competing interests

The authors declare that they have no competing interests.

## Authors' contributions

LCEP participated in the design and coordination of the study, interpreted the data, and performed the drafting of the manuscript. EM conceived of the study, participated in its design and coordination, interpreted the data, and performed the drafting of the manuscript.

SF participated in the design and coordination of the study, developed its questionnaire, and critically revised the manuscript. MU participated in the drafting of the manuscript, performed the statistical analysis, and interpreted the data. MC and TM participated in the statistical analysis, and revised the final draft of the manuscript. MW conceived of the study participated in its design and coordination, interpreted the data, and critically revised the manuscript. All of the six authors were equally involved in reading and approving the final manuscript.

## Pre-publication history

The pre-publication history for this paper can be accessed here:

http://www.biomedcentral.com/1472-684X/9/16/prepub

## Supplementary Material

Additional file 1 **Declaration of non-participation**. This file contains the declaration of non-participation for people who did not want to participate in our study. Click here for file

Additional file 2 **Extract from the EPACS-questionnaire**. This file contains several questions from the EPACS-questionnaire, covering cause of death and illnesses, type of care, and quality of outpatient care at home. Click here for file
