# Supplementary material for: End-of-life care in Germany: Study design, methods and first results of the EPACS study (Establishment of Hospice and Palliative Care Services in Germany)
Source: BMC Palliat Care. 2010 Jul 30;9:16. doi: 10.1186/1472-684X-9-16 (PMC2921359; doi:10.1186/1472-684X-9-16)
Supplement: Additional file 1 — Declaration of non-participation. This file contains the declaration of non-participation for people who did not want to participate in our study. [file 1472-684X-9-16-S1.DOC]

|  |  |
| --- | --- |

**Declaration of non-participation**

| □ | I don’t want to participate because | |
| --- | --- | --- |
|  | □ | the topic is too emotionally draining. |
|  | □ | I don’t have the time. |
|  | □ | I don’t participate in surveys in general. |
|  | □ | Other reason (please name): |
|  |
