# Supplementary material for: End-of-life care in Germany: Study design, methods and first results of the EPACS study (Establishment of Hospice and Palliative Care Services in Germany)
Source: BMC Palliat Care. 2010 Jul 30;9:16. doi: 10.1186/1472-684X-9-16 (PMC2921359; doi:10.1186/1472-684X-9-16)
Supplement: Additional file 2 — Extract from the EPACS-questionnaire. This file contains several questions from the EPACS-questionnaire, covering cause of death and illnesses, type of care, and quality of outpatient care at home. [file 1472-684X-9-16-S2.DOC]

|  |  |
| --- | --- |

**Extract from the EPACS-questionnaire**

II. Cause of death and illnesses

| **4.** | **Was your relative’s death related to a progressive, incurable, lethal illness?** (e.g. cancer, dementia, Alzheimer‘s disease or a chronic heart disease)**?** | |
| --- | --- | --- |
|  | □ yes | □ no |

| **5.** | **Where did your relative die?** | | | | | | | | | | | | | | | | | | | | | |
| --- | --- | --- | --- | --- | --- | --- | --- | --- | --- | --- | --- | --- | --- | --- | --- | --- | --- | --- | --- | --- | --- | --- |
|  | □ | At home | | | | | | | | | | | | | | | | | | | | |
|  | □ | **Intensive care unit** at the hospital | | | | | | | | | | | | | | | | | | | | |
|  | □ | **Palliative care unit** at the hospital (ward for the supply of incurable ill patients in the last weeks of life) | | | | | | | | | | | | | | | | | | | | |
|  | □ | **Normal ward** at the hospital | | | | | | | | | | | | | | | | | | | | |
|  | □ | In a nursing home | | | | | | | | | | | | | | | | | | | | |
|  | □ | In a hospice | | | | | | | | | | | | | | | | | | | | |
|  | □ | Somewhere else  **(please name):** |  |  |  |  |  |  |  |  |  |  |  |  |  |  |  |  |  |  |  |  |
|  |  |  |  |  |  |  |  |  |  |  |  |  |  |  |  |  |  |  |  |

| **6.** | **Did your deceased relative suffer from one of the following illnesses in the last four weeks prior to his/her death?** *(Several answers possible.)* | | | | | | | | | | | | | | | | | | | | | | | | | | | | | | | | | | | | |
| --- | --- | --- | --- | --- | --- | --- | --- | --- | --- | --- | --- | --- | --- | --- | --- | --- | --- | --- | --- | --- | --- | --- | --- | --- | --- | --- | --- | --- | --- | --- | --- | --- | --- | --- | --- | --- | --- |
| ! | In order to be able to compare the supply concerning different illnesses it is important for us to know which illnesses your relative suffered from.  For data protection reasons you should only answer this question if this would not have been against your deceased relative’s will. | | | | | | | | | | | | | | | | | | | | | | | | | | | | | | | | | | | | |
|  |  | | | | | | | | | | | | | | | | | | | | | | | | | | | | | | | | | | | | |
|  | cancer | | | | | | | | | | | | | | | | | | | | | | | | | | | | | | | | | | | | |
|  |  | □ | Breast cancer | | | | | | | | | | | | | | | | | | | | | | | | | | | | | | | | | | |
|  |  | □ | Lung cancer | | | | | | | | | | | | | | | | | | | | | | | | | | | | | | | | | | |
|  |  | □ | Cervical cancer | | | | | | | | | | | | | | | | | | | | | | | | | | | | | | | | | | |
|  |  | □ | Prostate cancer | | | | | | | | | | | | | | | | | | | | | | | | | | | | | | | | | | |
|  |  | □ | Bowel cancer | | | | | | | | | | | | | | | | | | | | | | | | | | | | | | | | | | |
|  |  | □ | Other type of cancer  **(please name):** | | | |  | |  | |  | |  | |  | |  | |  | |  | |  | |  | |  | |  | |  | |  | |  | |  |
|  | |  | |  | |  | |  | |  | |  | |  | |  | |  | |  | |  | |  | |  | |  | |  |
|  | □ | stroke | | | | | | | | | | | | | | | | | | | | | | | | | | | | | | | | | | | |
|  | □ | dementia (e.g. Alzheimer‘s disease, age dementia) | | | | | | | | | | | | | | | | | | | | | | | | | | | | | | | | | | | |
|  | □ | Chronic severe illness of the nervous or muscle system (e.g. multiple sclerosis, amyotrophic lateral sclerosis; **not:** stroke) | | | | | | | | | | | | | | | | | | | | | | | | | | | | | | | | | | | |
|  | Non-malignant, chronic severe organ illness like: | | | | | | | | | | | | | | | | | | | | | | | | | | | | | | | | | | | | |
|  |  | □ chronic severe heart disease (including myocardial infarction) | | | | | | | | | | | | | | | | | | | | | | | | | | | | | | | | | | | |
|  |  | □ chronic severe kidney disease | | | | | | | | | | | | | | | | | | | | | | | | | | | | | | | | | | | |
|  |  | □ chronic severe lung disease | | | | | | | | | | | | | | | | | | | | | | | | | | | | | | | | | | | |
|  | □ | Diabetes | | | | | | | | | | | | | | | | | | | | | | | | | | | | | | | | | | | |
|  | □ | AIDS | | | | | | | | | | | | | | | | | | | | | | | | | | | | | | | | | | | |
|  | □ | I don’t know | | | | | | | | | | | | | | | | | | | | | | | | | | | | | | | | | | | |
|  | □ | Other severe diseases:  (bitte nennen): | |  |  |  | |  | |  | |  | |  | |  | |  | |  | |  | |  | |  | |  | |  | |  | |  | |  |  |
|  |  |  | |  | |  | |  | |  | |  | |  | |  | |  | |  | |  | |  | |  | |  | |  | |  |  |

IV. Type of care

| **12.** | **Where and by whom has your relative been cared for in the last 4 weeks prior to his or her death?** | | | | | | | | | | | | | | | | | | | | | |
| --- | --- | --- | --- | --- | --- | --- | --- | --- | --- | --- | --- | --- | --- | --- | --- | --- | --- | --- | --- | --- | --- | --- |
|  | □ | At home by family and / or friends | | | | | | | | | | | | | | | | | | | | |
|  | □ | At home by a nursing service | | | | | | | | | | | | | | | | | | | | |
|  | □ | At home by a specialized palliative care nursing service | | | | | | | | | | | | | | | | | | | | |
|  | □ | At home by a community hospice service | | | | | | | | | | | | | | | | | | | | |
|  | □ | At the hospital in an **intensive care unit** | | | | | | | | | | | | | | | | | | | | |
|  | □ | At the hospital in a **palliative care unit** (ward for the supply of incurable ill patients in the last weeks of life) | | | | | | | | | | | | | | | | | | | | |
|  | □ | At the hospital in a **normal ward** | | | | | | | | | | | | | | | | | | | | |
|  | □ | At a nursing home | | | | | | | | | | | | | | | | | | | | |
|  | □ | At an inpatient hospice | | | | | | | | | | | | | | | | | | | | |
|  | □ | others  **(please name):** |  |  |  |  |  |  |  |  |  |  |  |  |  |  |  |  |  |  |  |  |
|  |  |  |  |  |  |  |  |  |  |  |  |  |  |  |  |  |  |  |  |
|  | □ | I don’t know | | | | | | | | | | | | | | | | | | | | |

V. Quality of outpatient care at home

Please rate the quality of care your relative has been received by professionals at home (outpatient care) in the last four weeks prior to his or her death.

| **14.** | **For how long has your relative been cared for by a nursing service in the last four weeks prior to his or her death?** | | | | |
| --- | --- | --- | --- | --- | --- |
|  | □ | Not at all |  | □ | 2 to less than 3 weeks |
|  | □ | Less than a week |  | □ | 3 to 4 weeks |
|  | □ | 1 to less than 2 weeks |  | □ | I don’t know |

| **15.** | **Professional’s Reachability: To which extend do you agree with the following statement concerning your relative’s care by specialists at home?**  In urgent circumstances the following professionals were always easily reachable (e. g. also at night or at the weekends): | | | | | | | |
| --- | --- | --- | --- | --- | --- | --- | --- | --- |
|  |  | **Has not been involved** | **Professional has been involved  I … agree** | | | | | **I don’t know** |
|  |  |  | **totally** | **rather** | **partly** | **rather don‘t** | **totally don‘t** |  |
|  | Physician | □ | □ | □ | □ | □ | □ | □ |
|  | Nursing service | □ | □ | □ | □ | □ | □ | □ |
|  | Staff member of community hospice service | □ | □ | □ | □ | □ | □ | □ |

| **16.** | **Information and time: To which extend do you agree with the following statement concerning your relative’s care by specialists at home?**  **a)** The following professionals had enough time if my relative needed them: | | | | | | | | | |
| --- | --- | --- | --- | --- | --- | --- | --- | --- | --- | --- |
|  |  | **Has not been involved** | **Professional has been involved  I … agree** | | | | | | | **I don’t know** |
|  |  |  | **totally** | **rather** | | **partly** | | **rather don‘t** | **totally don‘t** |  |
|  | Physician | □ | □ | □ | | □ | | □ | □ | □ |
|  | Nursing service | □ | □ | □ | | □ | | □ | □ | □ |
|  | Employee outpatient hospice service | □ | □ | □ | | □ | | □ | □ | □ |
|  | **b)** **Please only answer the following question, if your relative has been responsive in the last four weeks prior to his death:**  Information about … given by the **physician** was comprehensible and sufficient. | | | | | | | | | |
|  |  | | **I … agree** | | | | | | | **I don’t know** |
|  |  |  | **totally** | | **rather** | | **partly** | **rather don‘t** | **totally don‘t** |  |
|  | …therapies (effects, side-effects) | | □ | | □ | | □ | □ | □ | □ |
|  | …current health status | | □ | | □ | | □ | □ | □ | □ |

| **17.** | **Symptoms and Problems: To which extend do you agree with the following statement concerning your relative’s care by specialists at home?** | | | | | | | | |
| --- | --- | --- | --- | --- | --- | --- | --- | --- | --- |
|  |  | **Problem didn‘t exist** | | **I … agree** | | | | | **I don’t know** |
|  |  | |  | **totally** | **rather** | **partly** | **rather don‘t** | **totally don‘t** |  |
|  | My relative’s pain was treated sufficiently | | □ | □ | □ | □ | □ | □ | □ |
|  | Other physical symptoms (e.g. nausea, shortness of breath) were treated sufficiently. | | □ | □ | □ | □ | □ | □ | □ |

| **18.** | **Competence and help: To which extend do you agree with the following statement concerning your relative’s care by specialists at home?** | | | | | | | | | |
| --- | --- | --- | --- | --- | --- | --- | --- | --- | --- | --- |
|  | 1. The following professionals were helpful regarding emotional support: | | | | | | | | | |
|  |  | | **Has not been involved** | **Professional has been involved  I … agree** | | | | | **Deceased did not want support** | **I don’t know** |
|  |  |  |  | **totally** | **rather** | **partly** | **rather don‘t** | **totally don‘t** |  |  |
|  | Physician | | □ | □ | □ | □ | □ | □ | □ | □ |
|  | Nursing service | | □ | □ | □ | □ | □ | □ | □ | □ |
|  | Employee outpatient hospice service | | □ | □ | □ | □ | □ | □ | □ | □ |

| **22.** | **In a general way, how satisfied are you concerning your relative’s oupatient care at home?** | | | | | |
| --- | --- | --- | --- | --- | --- | --- |
|  | **Not at all** | **little** | **partly** | **rather** | **totally** | **No judgment possible** |
|  | □ | □ | □ | □ | □ | □ |

Thank you for your participation!
